# The credibility crisis and democratic governance: how to reform university governance to be compatible with the nature of science

**DOI:** 10.1098/rsos.220808

**Published:** 2023-01-25

**Authors:** Zoltan Dienes

**Affiliations:** School of Psychology, University of Sussex, Brighton, UK

**Keywords:** university governance, democracy, open democracy, open science

## Abstract

To address the credibility crisis facing many disciplines, change is needed at the institutional level. Science will only function optimally if the culture by which it is governed becomes aligned with the way of thinking required in science itself. The paper suggests a series of graduated reforms to university governance, to radically reform the operation of universities. The reforms are based on existing established open democratic practices. The aim is for universities to become consistent with the flourishing of science and research more generally.

## 1. Introduction

Many areas of science have been facing difficulties in credibility with a sense that the scientific process is not as healthy as it could be. There is low replicability of studies ([1], chapter 2), possibly associated with a failure of a field to self-correct [2]; and at the same time, there is a hyper-competitive culture aligned with perverse incentives that may reward substandard science [3]. The solutions to this credibility crisis will surely involve multiple levels of reform [4]. Specifically, cultural change is needed at the institutional level, which is the level that this paper addresses. Initially, a simple account of how knowledge grows is presented. Then a brief historical overview is provided of the growth of knowledge and its relation to how decisions are made in the broader social context. Classical Athens is taken as an example of matching between the governance of the society as a whole and the growth of knowledge. Next, the state of governance of UK universities is considered. Finally, open democratic practices that are Athenian-like and that have been tested in politics are considered for how a university might be governed in an open democratic way in order for the university to align itself with the way knowledge grows.

## 2. How does knowledge grow?

Popper [5] asked ‘How does knowledge grow?’ His answer in general, is by trial and error; try ideas out and see what works; reject those that do not work. How could that process be enhanced? According to Popper, traditionally schools of thought were passed down from master to disciple with an aim to impart a doctrine pure and unchanged. There is in such a tradition a hierarchy with roles filled by people by virtue of their characteristics: master and disciple. This is a good way of conserving knowledge as it is, but not for promoting the growth of knowledge. But consider an alternative, which Popper calls the critical tradition: the master says ‘Here is my idea to solve a problem; can you improve on it?’ It can be difficult to encourage others to do better than oneself. Thus, the critical tradition as a second-order tradition that is passed on from mentor to pupil has to be constantly fought for: businesses, religious leaders, politicians and even academics will regularly try to stamp out criticism of their ideas. The critical tradition occurs when there is a culture of considering arguments for their own sake, with small regard for the authority of the person stating them. That is, in a critical tradition roles are fluid, and what is important is the quality of ideas. Taking part in a critical tradition may be psychologically easier when people see ideas, theories and data as having an objective existence apart from themselves, with properties that must be discovered; this is what it means to consider arguments for their own sake. Then people can refute a theory without thinking they themselves have been harmed [6]; cf. also [7]. Let an open society be a society in which such a critical tradition is encouraged (cf. [8]). Let democracy be an open society in which there are institutions that encourage a critical tradition independent of any individual. Thus an autocratic ruler may promote an open society if that was the sort of thing that ruler liked; but the society would not as such be a democracy, because the existence of the open society would depend on the whims of a particular ruler.

## 3. Lessons from history

There is intriguing evidence of a historical relationship between the growth of knowledge and the existence of an open society, especially democracy. Popper [5] suggests how Thales, around 600 BCE, proposed a natural principle for how the world works, only for his apparent student, Anaximander to come up with something logically better, starting a critical tradition. For the next several hundred years in Athens, there was an astonishing flourishing of knowledge, in mathematics, astronomy, history, psychology and medicine. Knowledge and open critical discussion continued into the extended Greek and Roman world for some time AD, but the critical tradition gradually withered. For example, the *Epidemics* of the Hippocratic corpus (fifth century BCE) mainly indicated how their treatments failed ([9], chapter 5) in contrast to case histories from later centuries (consider the numerous triumphs Galen, fl. second century AD, described in outwitting other doctors ([10], e.g. chapter 7); after Galen, there were no students of his who tried to produce better solutions, at least not for many centuries). Almost exactly contemporaneously with the rise and decline of the flourishing of knowledge, there was a rise and decline in Athenian-style democracy. In Athens itself, the initial reforms of Solon (600 BCE) were strengthened by Cleisthenes (coming up to 500 BCE), then after further gradual refinements, Athenian democracy was formally ended in 332 BC by the conquest of Alexander the Great. However, as seen as part of an ecology of about 1000 Greek states, democracy robustly continued well into the second century BCE, with the number of democracies actually increasing for some time [11]. Over several hundred years from before until after the classic period, these Greek states showed shifting mixtures of democracies and elite control. Based on archaeological and historical evidence, Ober argued that cultural flourishing (and wealth) followed not the rise of conservative institutions but tracked the development of democratic rule-egalitarian institutions.

There was both an explosion of knowledge and the implementation of a robust long-lasting democracy during the time of classic Athens, followed after a delay by a slow stagnation in knowledge growth. At other times and places, when there was an open society, knowledge also flourished—even without democracy. Saliently, from 800–1400 Arabic science drew from Greeks and Indians, and made major progress [12]. There was not democracy, but absolute rule by Abbasid caliphs. Any given caliph might support an open society (e.g. initially especially Al-Ma'mun), in the sense of encouraging critical discussion of ideas (e.g. Al-Khalili [12], pp. 67–68). When a caliph, or others in positions of authority, supported the free exploration of ideas, knowledge grew; when a subsequent caliph was more conservative, learning shrank (e.g. [12], p. 230, cf. also p. 194). The relation of general openness during this time and scientific progress bears further investigation.

The critical tradition sustained in the Arab world eventually found its way to the medieval Italian states. From about 1100, these states were already exploring democratic governance, using mixtures of lot and election (e.g. [13,14]). The assembly politics often characterizing decision making in those states entailed considering arguments for their own sake even if the person voicing them may be of low rank. Thus, the ground was laid for exploring new ideas; and later there is indeed the outpouring of new forms in art, invention and later science. Ferris [15] picks up the story of the intertwining of democratic values and the growth in science in the 1600s onwards, noting how science developed in the most liberal countries, and how conversely scientists were key people pushing for democratic change. A further explosive growth in knowledge occurred alongside the progressive rejection of authoritarian political values and the development of liberal values, from the enlightenment onwards.

Yet the growth of knowledge has also occurred when society as a whole was not especially open, and conversely, science often did not occur where there were even democratic institutions. An example of the first point is the steady growth of knowledge throughout Chinese history. Up until about 1400, China was hundreds, and sometimes thousands of years, ahead of Europe in technological development. Needham [16] spent decades documenting how many innovations came from China, for example, China pioneered inoculations (possibly tenth century and certainly by 16th); China developed mechanical clocks six centuries before Europe. True, China throughout its history has valued scholars very highly and had a well-organized civil service based on exams rather than (explicitly) on pedigree. But throughout this time the emperor in principle had the final say on any matter (including an edict that held from 653 forbidding the private possession of astronomical instruments ([17], p. 228)), and there is little evidence of democratic processes (except briefly for a period in the Zhou dynasty, 1050–221 BCE ([14], p. 150)). There was the constant development of technology—but not the explosive development of science. Needham asked why did modern science not develop in China and only in Europe? Why did knowledge of the physical world grow steadily in China, and yet not explode like it did in Europe? Needham suggested that ‘There was no modern science in China because there was no democracy' [16, p. 152]. Needham pointed out that science is indifferent to who makes the argument; thus ‘these civilizations which have developed an … exaggerated respect for teachers, will have to modify it' [16, p. 140]. In sum, ‘there is a real kinship between the scientific mind and the democratic mentality, ‘ namely, skepticism, anti-authoritarianism, not letting others decide on aims or assessment of evidence, ‘a give and take, a live and let live attitude’ [16, p. 143]. (For a review of other hypotheses by Needham and others to address this question, see [17].)

Conversely, Stasavage [14] and Graeber & Wengrow [18] present historical, archaeological and anthropological evidence for democracy, in the sense of decision by assemblies, being a common solution to the problem of political governance, and often in large-scale societies. If that is true, why did science not emerge multiple times? Debates in assemblies, to the extent criticism of any individual's views are welcome and not just tolerated, promote a critical tradition. And a critical tradition allows knowledge to grow, but it need not be specifically scientific knowledge. Graeber & Wengrow argue for the political sophistication of the indigenous Americans, whose skills were honed in assembly politics, and who could hold their own if not best European intellectuals of the 1600s in political and social arguments. Indeed, according to Graeber and Wengrow, native American arguments may have, unacknowledged, transformed the course of European intellectual history. Consider also the democratic politics in India at the time of the start of Buddhism (Mahaparinibbana sutta in [19]), which occurred contemporaneous with the development of ideas about the mind which continue to influence modern thinking (e.g. [20]). Clearly there is no deterministic relation between science and democracy; but there is synergy. The lesson to draw from history is that when science and democracy occur together, science is facilitated. Science might happen without democracy, and democracy without science—but put them together and see what happens.

## 4. Athenian-style democracy

**Figure 1.**
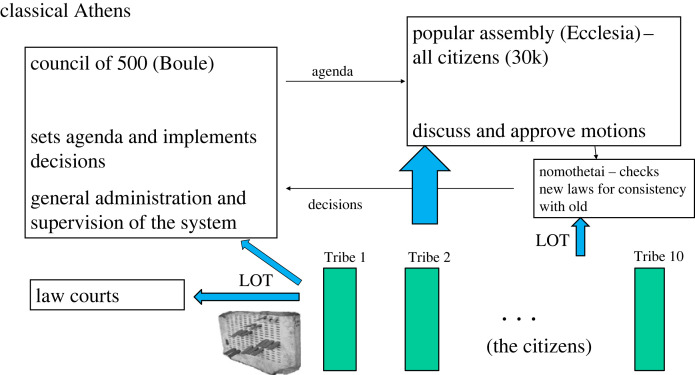
Sketch of classic Athenian democracy. In Athens circa fourth century BC, eligible citizens were assigned by lot, i.e. randomly, to the main governing bodies and the law courts. Of about 1000 posts, 90% were determined by lot and the rest by election. The majority of citizens would have spent at least a year at some point in their life in the main governing body, the boule. Some form of such governance lasted for hundreds of years in Athens and other Greek states.

Athenian democracy emphasized the selection of arguments that could in principle be provided by anyone, rather than primarily the selection of people according to their traits as such [21]. Let us consider some details, because they will be useful for considering modern reform (for a readable overview with the specific aim of implementing the principles in modern corporate institutional governance, see [22]; see also [23,24]. Athenians were divided into 10 tribes pooling different types of citizens (urban, rural, coastal) into in-groups to unite pre-existing cultural divisions (consider the use of houses or colleges in some universities). Day-to-day business was organized by the Council of 500 (the boule), consisting of 50 eligible citizens selected by random lot from each tribe for a period of a year. Each month the 50 from one tribe would set the agenda for the business of the day, and prescribe the meetings of the Assembly (which we will come to). Other duties include overseeing the work of all officials and financial oversight. Each day officials from the other tribes in the Council make decisions concerning the agenda. The leader of the Council was selected daily from the tribe in charge of agenda-setting that month: one person was the nominal head of state for one day alone! In sum, central decision-making was deliberately integrated over many people, chosen by lot from the citizenship. Roughly weekly, the citizens, or the subset who turned up (maybe a fifth of all citizens on any one occasion), formed the Assembly (ecclesia). The agenda was set by Council, and in principle any citizen could speak on any motion, before a vote to decide each proposal. One further institution is worth mentioning: the nomothetai. This was a panel formed by lot from eligible citizens to reflectively consider arguments for and against proposed general laws before a final decision was actually made to accept them. We will draw on this important institution later. Figure 1 for a sketch of the overall structure of Athenian democracy.

## 5. The state of UK universities

Coming into this millennium, many UK universities were democracies at the school (i.e. faculty) level. School meetings were decision-making bodies, with decisions made usually by majority vote. Decisions fed up to the central university level. Senior management at the central level then had to do their best to render coherent at the institutional level the way these parts fitted together. Subsequently, in about the first decade of the millennium, many UK universities moved to more or less complete non-democratic top-down control, where senior management made decisions, and the Deans of schools were to work out how to implement those decisions to management's satisfaction. The Dean rather than faculty had final say at school level. The Vice-Chancellor (VC) was appointed by Council with no involvement by faculty at large (with the members of Council being appointed by Council). These reforms occurred in the tradition of ‘New Public Management’, a philosophy of public sector management which started to be implemented in the Thatcher years, and has become dominant in the UK in its higher education policy [25–27]; see [28], for a history of UK education sympathetic to this approach).

In many universities in the UK, top-down control is exerted by management, who are perceived to be a class separate from academics with no real accountability [29]. These managers may apply strong pressure on academics to achieve key performance indicators. The result is managerialism: the worth of an academic (for getting jobs, promotion, respect) is determined by set performance goals, typically including getting grants and publishing in high-impact-factor journals. The first duty of a researcher is not high-quality research by their own judgement, but to fulfil the agenda of an increasingly large class of managers (whose agenda is to boost e.g. world rankings and other metrics). It is not obvious that top-down control by senior managers is the best way of dealing with a rapidly changing and unpredictable environment that is necessarily what the interface of knowledge and ignorance consists in. For the failure of complete top-down control in project management in unpredictable environments for aid agencies, see Honig [30]; and its failure in the secondary education sector, Honig [31]. Honig [31] presents evidence that top-down management with close monitoring and control backfires when those managed are already there because they want to be: such management produces both selection effects, the loss of good people, and motivational changes in those that remain (for the latter see also [32], chapter 5). Martin, reviewing the history of UK university governance with an eye on the management literature, asks, ‘Why, when the management literature of the last two decades has stressed the benefits of flatter organizational structures, of decentralization and local initiative … have many universities been intent on moving in precisely the opposite direction of greater centralization with a more hierarchical, organizational structure, top-down management … and ever more cumbersome and intrusive procedures?' [27 p. 7].^1^ And of specific relevance to science, Xu *et al*. [38] found that scientific teams with a flat rather than hierarchical structure produced more novel ideas and a higher long-term citation impact.

Attempting to incentivize ‘performance’ when what really counts as performance cannot be easily measured—as is the case in attempting to understand the unknown—will generally backfire [30,39]. Simple-minded performance targets famously backfire even for simple problems. Consider the rat tails of Hanoi. At the beginning of the twentieth century the French colonial rulers of Hanoi wanted the city rid of rats. To bring on board the local population, money was offered for each rat tail delivered as proof of a killed rat. Yet the rats only increased. It turned out the locals, being resourceful, set up rat farms [40]. When an expert needs to exercise judgement in an unpredictable environment, sustained incentives to maximize something just because it is measurable, typically distort best practice. The best person to judge strategy and tactics for dealing with a research problem is the researcher themselves. Incentivizing them to, for example, apply for grants, will distort how time is best spent. If a researcher needs a grant to further their research, they have no need of a manager to tell them to get a grant. Conversely, if their time would be better spent writing up that file drawer of papers, incentivizing them to apply for grants only promotes inefficiency. But the problem may be far worse than this. Key performance indicators filtering down from senior management as pressure on individual researchers may not just waste time—it may damage the integrity of science. It may produce, in effect, rat farms.

In simulation studies, Smaldino & McElreath [41] consider a population of different laboratories who differ in the degree of *p*-hacking they engage in while competing for grants. Given reasonable assumptions, those laboratories that *p*-hack the most are most successful in obtaining grant money—and thereby produce more progeny laboratories (via the PhD students and post docs they train) which carry on the same culture as the parent laboratory. The current environment of intense competition for grant money plausibly promotes low-integrity science (while of course being consistent with some successful laboratories that do value rigour, as shown, for example, by their commitment to open science). Smaldino *et al*. [42] consider solutions. In their simulations, the way to break the effect of the selection of *p*-hackers, was to borrow an idea from classical Athens, selection by lot: award grants by random lottery for those submissions that passed a minimal standard of methodological rigour. (Such a procedure not only can restore integrity, it also ensures a lack of discrimination based on gender, race, or institution in grant allocation.^2^) While the concept of *p*-hacking is not relevant to all disciplines, the argument plausibly generalizes for any similar process where cutting corners to the detriment of the quality of the research nonetheless allows outcomes that look convincing.

Satisfying key performance indicators typically involves publishing in journals with high impact factors. Often high-impact-factor journals are run by for-profit companies charging high publishing fees. A principle of how science should function is that anyone should be able to contribute according to only the quality of their contribution. High publishing fees mean that only those able to join a rich person's club, at an intuitional or individual level, can contribute. This undermines the proper functioning of science [45]. And it may be worse than that.

Does pressure to publish in high-impact journals produce *p*-hacked and less reproducible research? Direct evidence for pressure to publish producing poor quality publications as a general relationship is not yet in (consider the attempt by [46]). But there are some specific connections. Cash bonuses for publishing in high-impact-factor journals, as has been practised in Australia and China, is associated with retractions of papers [46]. Further, Fang & Casadevall [47] found a proportional relation between retractions and journal impact factor; most of these retractions were due to fraud [48]. While the retraction–impact factor relation may in part be because articles in high- rather than low-impact-factor journals are subject to greater scrutiny, this explanation is ruled out by Brembs [49] in his investigation of research quality across a range of scientific disciplines. He found a negative relation between journal impact factor and various measures of methodological rigour^3^ (also recently found by [51], in management science; and [52] found weakly negative relations in behavioural science and neuroscience). So if the papers are less good methodologically on average, why do they get published in higher impact-factor journals? Presumably because the authors were good at selling their results. In sum, managerialism at university level, situated in a dysfunctional ecosystem, selects for *p*-hacking/corner-cutting salespeople.

How is the rise of managerialism experienced by university staff? Shattock & Horvath [53] conducted extensive onsite interviews with staff at all levels of each university, at UK universities that spanned a wide range of rankings, to explore staff experiences. They found widespread dissatisfaction. ‘The sense that conditions for the pursuit of high quality academic work have worsened and are continuing to worsen is widespread, even in institutions that are most obviously successful. Criticisms that universities have become too top-down in their governance, and are insufficiently bottom-up, that good academic work is stifled by over-regulation and bureaucracy, and that too much academic business is handled by non-academic professionals, are commonplace’ [53, p. 104]. Similarly, Erickson *et al*. [29] in a survey of 5888 academic staff in the UK higher education sector, found only 10% of university staff were satisfied with senior management.

The question is, in terms of university governance, could we be doing better? In the next section we consider democratic solutions.

## 6. Open democracy

Hierarchical university governance may contribute to damage to scientific integrity. So what mode of governance might actually promote the growth of knowledge? That is, what way of governing universities would be consistent with a culture of the critical tradition, the tradition of carefully considering and selecting arguments rather than people? Arguments concerning the running of an institution can only be considered as such, by anyone with a stake in them, if there is transparency of information. Relevant information must be readily accessible (just as is required for science to function). What organizational structure produces transparency in a way that is useful?

Consider the following assumptions:

(1) Each academic has information about how the university is working.
(2) Different people have different relevant information.
(3) Information will be expressed when a person has to use it to make decisions.
(4) Those decisions will make best use of the information if people making decisions have to live by them.
(5) To select the best ideas (that integrate information well), ideas should be selected for, not people.

Ways of governing that satisfy these assumptions include those of Athenian-like democracy that we considered earlier. Athenian-like democracy has inspired a number of open democratic practices that have been explored in the political context in the last few decades [54]. We will review some of these practices now.

Before describing concrete ideas, consider an objection. Given the complex environment in which universities now operate, don't we need decisions made not democratically by the uninformed but rather by experts (cf. [55]), with a top-down governance structure that thus allows nimble and agile decisions? As against this, if the above five assumptions are accepted, some form of democracy may foster decision-making where the most information is maximally integrated in the time available^4^: structures embedding such practices should produce networks between people closer to small-world networks than a strict top-down hierarchy could; and such networks allow more global integrated information [57]. Open democracy can ensure decisions are made by the well informed, as we discuss. And it is rare to hear modern universities described as nimble and agile [29,53]. Open democracy also presents a smorgasbord of ways of being democratic with different time scales of operation. Indeed, in Athens, important decisions were sometimes made or reversed very quickly, even in the space of a day ([22], e.g. pp. 133–134). Sometimes decisions should be fast, sometimes slow and reflective. With this in mind, let us see what is on offer.

### 6.1. The deliberative poll

The deliberative poll was developed by Fishkin [58,59], and will be used to illustrate the more general class of mini-publics, procedures by which people are selected from the population of citizens to deliberate an issue—such as citizen's assemblies, citizen's juries or citizens’ initiative reviews (which we will consider below) (see [60], for a review). Consider a difficult issue that concerns a community, and for which a reflective and informed decision is needed that takes into account diverse viewpoints—such as Brexit, immigration or, in a university setting, the principles for allocating resources to schools in a context of shifting student demand. Randomly select 200–300 people from the total population (one may decide to over-represent certain groups of people particularly affected by the issue). Given the selection is approximately random, everyone has a chance to contribute and no one is selected simply because they have a vested interest. The total sample is allocated into groups of 15 to discuss the chosen issue. The discussants are given information packs, each pack prepared by experts or protagonists of different views. The discussions are moderated to encourage everyone to contribute more or less equally, and for debate to focus on arguments *per se*. There is an opportunity for discussants to ask a panel of experts any questions that remain unresolved. After several meetings, discussants anonymously vote: as Fishkin puts it, the vote is then what the people would think if they had reflected. The deliberative poll has been used in many countries over the last few decades, for example in the UK, Europe, Australia, China, the USA, Canada and Mongolia (for critical discussion see [61], chapter 3; [60], chapter 3).

To take an example, in Ze Guo Township in China, the issue was how to spend the council's money [58]. Thirty projects were listed by the council. Two hundred and seventy-five citizens were randomly selected; of these 235 completed the poll, so the final sample was close to random. Fishkin provides evidence that participants became more informed as a result of the discussions, that there was little to no domination by privilege, that there was little to no group polarization, and that priorities shifted toward projects that would benefit the whole town. Of course, the devil would be in the details to get such good procedural outcomes (e.g. well-trained moderators). People chose a sewage treatment plant, park and a main road; not, for example, a fancy town square. These choices surprised officials but they acted on the results of the poll.

A minipublic can be used to either set the agenda of a committee, or to make decisions on issues defined by a committee, and both roles may be useful in a university. To adapt the minipublic to a university, one might adjust the number of people selected, or the length and number of meetings, according to the issue considered. Note the similarity of the minipublic in finalizing decisions to the nomethetai in the Athenian model.

### 6.2. Participatory budgeting

Engaging the community as a whole in planning budgets was pioneered in Porto Alegre in Brazil in 1989 onwards in a procedure called participatory budgeting (see [60], for an introduction). In this case, neighbourhood assemblies voted locals to represent the neighbourhood for a year in a local committee to plan projects and their priorities. People were also elected to one of several thematic committees (education, transport, etc.) to plan projects and their priorities for each theme. Locals from each neighbourhood and thematic assembly were also voted to an overall coordinating central committee. The central committee decided projects and allocated a substantial proportion of the council's budget. The committees also involved people with relevant technical and financial expertise. A person could be elected for no more than two terms. The choice of local people, the shortness of the term and the limited number of terms per person is what roughly corresponds to random sampling in deliberative polls in the sense of being the mechanism that limits entrenchment of certain people in decision making. Participatory budgeting was regarded as so successful it has been taken up in different forms in more than 2700 governments (though in 2017, participatory budgeting was suspended in Porto Alegre itself [62].

For a university following the Porto Alegre model, schools could, as local neighbourhoods, select faculty on a rotating basis to a school budgeting committee for a year to decide school projects. Staff from relevant groups could be selected on a rotating basis to committees devoted to certain themes at institutional level, such as IT, catering, grounds, etc. Similarly, each school could select on a rotating basis two representatives for a central budgeting committee to determine institution-wide spending and to finalize decisions of the other committees. In the participatory budget model, selection is by local election; but people could be selected (semi) randomly.

### 6.3. The citizens’ initiative review

The citizens’ initiative review [63] is a one-group minipublic where the group jointly summarizes the best arguments pro and contra a proposal, and also summarizes how they voted. The aim is to provide an information leaflet for a referendum on the proposal that reflects the range of views ordinary people would have if they reflected on the issues and made themselves informed. Crucially, therefore the information leaflet is not provided by vested interests. The citizens review initiative has been used extensively in the state of Oregon, USA, where studies indicate that voters appreciated the information and were better informed as a result of it.

### 6.4. Allocation of citizens to roles

Democracy is often associated with voting, as that is how our current representative democracies work. But when academics vote for people in the university to be on senate, council or some other position, often the vote is based on limited information, such as what school they work in, or what other committees they have been on. The facts given may be of marginal relevance to how that person would contribute to that role. And if the information is enough to be seen as relevant, voting is a mechanism for selecting people who stand out from other people, in other words, for selecting elites [13]. Having the resources or motivation to promote oneself in an election is not the same thing as having the qualities that would make one good at the job the election is for. The medieval Italian city states realized this. But they also thought selection strictly by lot may not select the best people for the job, even if it ensures the top jobs are not all held by people of a certain class. So these city states devised various combinations of election and lot to try to obtain the benefits of both procedures (see [64], chapter 7, for a systematic analysis of the possibilities). For example, one could select by lot a group of candidates, who are then chosen by election. An institution could decide what combination of processes will assign people to posts. One counter-defence of selection by lot alone, i.e. without election, is that the repeated use of such a procedure educates the citizenship; further if each person is merely a constraint in a global process of selecting ideas (as in science) the notion of selecting the right person may lose some of its relevance. Note that once people have been selected for the executive committee by an open democratic process, such a committee can in principle act in real time as fast as any committee now, depending on details.

## 7. Reforming the university

**Figure 2.**
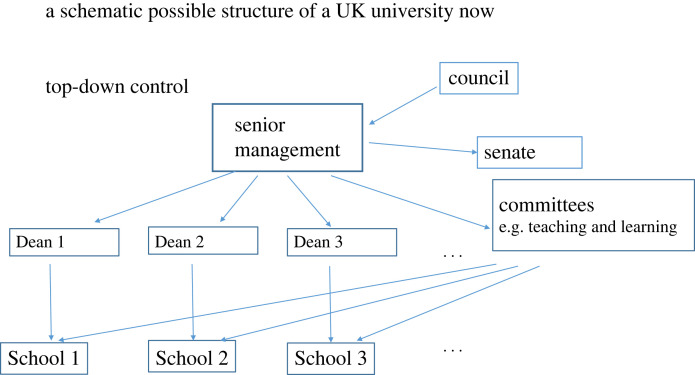
Schematic diagram of top-down governance as might exist in a current UK university. Control runs from top to bottom. Senate allows some pushback on academic matters but senate may only in practice have the power for suggesting that senior management or council reconsider.

**Figure 3.**
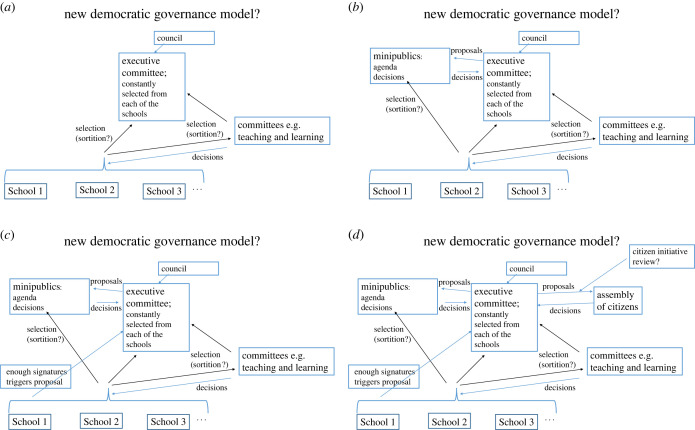
A series of democratic changes to the governance structure shown in figure 2. (*a*) The same command flow through committees could be kept as in figure 2, but people assigned to committees by sortition (lot). (*b*) The executive committee could use minipublics to set certain agendas or make decisions. (*c*) To this could be added a standing right for decisions to be reviewed by the executive committee if a petition with enough signatures is submitted. (*d*) There could also be a general assembly of citizens to which the executive committee could submit some decisions. So that the assembly can make an informed choice, such referenda could be supported by citizen review initiatives.

Figure 2 shows a schematic summary of the flow of control from the top down in a hypothetical modern university. The simplest way of seeing how radical reform could be made is keeping the same structure but assigning people to roles by an open democratic process (figure 3*a*). Just as in Athens, assignment by lot does not mean there are no restrictions. Some jobs may be open only to senior lecturers or above for example. Or some committees may require experience of other committees.

While this one change is structurally simple, it is of course a radical first move. It involves the abolishment of senior management. Some people, notably senior management, might think this a possibly catastrophic first move. This possible first move is presented to illustrate how one can keep other things the same, yet radically change the democratic nature of governance to be similar to the style of governance that once thrived in a complex nation for over a hundred years. In practice, open democracy should be explored in small steps. One could first of all set up a minipublic, with commitment from senior management to abide by its decisions, on an issue of importance that needs time to consider, for example, the university's response to proposed pension reforms. The issue needs reflection and requires becoming immersed in relevant information, and should not be decided solely by people with one type of axe to grind. Figure 3*b* illustrates the addition of minipublics. Once their use has been explored and finessed, more changes could be explored.

Figure 3*c* shows a further step that could be taken as an initial small step. To increase recurrent information flow through the system, so that it functions as close as possible to a self-organizing dynamic system that maximizes global constraint satisfaction, if enough signatures are obtained for a petition, the executive committee could be obliged to reconsider a decision, then provide information for why they kept it or changed the decision. Finally figure 3*d* shows the addition of an assembly of citizens. On some occasions the executive committee may wish decisions be decided by referenda. In this case, citizens’ initiative reviews should be used to provide unbiased information about what is at stake. Whether an institution makes any one of the changes suggested in this sequence should be a slow process of exploration.

## 8. Conclusion

This paper outlined some broad principles for making decision making in a university more closely match decision making in science, arguing both that open democracy allows good decisions (else why do we use it in science, one of our most successful endeavours?); and that good science will be promoted when embedded in a broader culture that operates in the same way as itself. But much was left unaddressed. Who counts as a citizen? That will need to be addressed by an institution (bearing in mind we should be accountable to students, in a way we are not in the current system). What about professional services? Just as politicians need a civil service that has expertise and will offer alternative proposals, so academics need professional services to allow the university to run. The organization of professional services has not been addressed, but presumably some of the same principles could apply to their governance. What about Council, the ultimate governing body of a university? Shattock and Horvath ([53], p. 100) are scathing about how Councils are currently formed in terms of what is expected of them. An institution will need to decide how best to make Council better informed and more accountable through open democratic practices.

The argument is not at all that the credibility crisis in science, and the current dissatisfaction with university governance, is the fault of any particular individuals, most of whom are simply people trying to make the best decisions. The problem is the structure in which senior management operate. No amount of focus groups to determine key catch phrases to repeat as slogans will change that. The current governance structure is almost designed to be divisive and demoralizing. Just like an Abbasid caliph, a current senior manager or VC of a modern university may somehow benignly run a happy and smooth operation that promotes the flourishing of knowledge despite the system. Until the next VC comes along. Let's make the system itself work.

One concern is whether open democracy will increase the admin load on people. If committees maintained the same numbers as currently, the average committee load ceteris paribus remains the same. There is an incentive difference, however, that may reduce average committee burden: while professional bureaucrats have an incentive to maximize number of committee meetings (are there not more data on key performance indicators and ‘academic drivers’ to be drilled down into?), people who are committee-averse have an incentive to reduce them. On the other hand, deliberative polls formed to consider an issue will increase admin time. The trade-off is that this increase in time is time spent being informed about how things work, being a part of its workings, and making a difference to how things work. A related concern is random lot means people may be selected who consider they are already being overworked. One could be allowed to refuse such an assignment a certain number of times in a certain period. But in the end the system will only function if people are committed to being citizens. The current pay of senior management could be split among citizens, and people could opt out of being citizens. People who opt out would not be taken seriously when they complained bitterly about decisions that were made.

But is democracy weak under pressure, inherently irrational, maybe susceptible to mob rule? Tangian ([65], p. 294) argued that Athenian democracy would make unstable decisions for ‘situations close to controversy [i.e. 50/50 for and against in the population] because a negligible disturbance results in a significant change. At the controversial point, the decisive majority opinion, whether positive or negative, depends on the positions of just a few individuals.’ Similarly, there are long-standing theorems, such as Arrow's theorem, that seem to show a group of people making decisions by voting are necessarily irrational in one way or another. Deutsch ([66], chapter 13) points out that these arguments, that might be used against democracy, including Tangian's just mentioned, presume there are fixed options regarded by people with fixed preferences. But the point of critical discussion is to change one's preferences and allow new options to unfold. That is the real substance of decision making. Open democracy allows preferences and lists of options to change in a rational way. And to the extent that having people form small minipublics with moderated discussion promotes sensitivity to reason, democracy need not amount to mob rule.

How could these reforms be implemented in practice? Popper [67] argued for piecemeal social engineering, that is taking small steps at any one time, as the politician is aware that the perfect state, if attainable, is far distant, and each change along the way will have unintended consequences, which are best dealt with one by one. Could a university VC be persuaded there is at least one decision that is important for people, yet one they could be willing to give up to a minipublic? It is sometimes in a leader's interest to have handed over difficult decisions to a public body, such the Citizens’ Assemblies to consider abortion or marriage equality in Ireland [68]. Or if a VC genuinely considers a pension deal is the best one for the university, and they trust fully informed rational discussion will lead to the same conclusion, why not, by having a minipublic decide, save themselves from losing goodwill for the rest of their term? Or why not start with a decision about which they have no axe to grind, but staff do care about—might that not help staff morale? Might not a university who started along this path become a beacon for other universities, be the one that stood out from the other sheep by not copying their nearest neighbour sheep? Start with a single decision. Then finesse the procedure to make it better.

Gradually finessing the procedure will take effort; but as indicated there are reasons to think it is worth the effort. In fact, open democracy may uniquely solve a key problem. Fukuyama [69] argued that all political systems face the problem of elite entrenchment bringing about inevitable decay of the system. Analogous arguments may apply to universities, not just nation states, when there is a limited pool of managers who, schooled by the same system, approach problems in similar ways. Despite what Fukuyama claimed, there is a way of hindering elite entrenchment: by constantly randomly selecting citizens to act as decision makers, there is a constant input of new viewpoints. Injections of randomness are necessary for creativity. Of course, people with many good ideas may still be especially influential. The goal is to make sure that in taking on board ideas, what matters is the quality of the ideas. Thus, good ideas should be selected, whatever their source.

The benefits of open democracy may also be motivated in the light of McGregor's [70] influential distinction between two theories that management might have about the psychology of people. According to Theory X, ‘the average human being has an inherent dislike of work … [and so] must be coerced, controlled, directed, threatened with punishment to get them to put forth adequate effort … ’ (p. 43). By contrast, according to Theory Y, ‘The expenditure of physical and mental effort in work is as natural as play or rest … [a person] will exercise self-direction and self-control to the service of objectives to which [they are] committed … the capacity to exercise a high degree of imagination, ingenuity and creativity in the solution of organizational problems is widely, not narrowly, distributed … ’ (pp. 59–60). A relevant principle may be that when management treats people in ways that express certain expectations of them (e.g. Theory X or Y), management tends to get what it expects (for a business case study, see [71]). Likewise, requiring academics to fulfil narrow key performance indicators, may tend to produce academics who do as they are directed (and no more). Managers who subscribe to Theory X may regard this as proof of their position—even as science and organizational creativity suffers. Yet trusting faculty to solve important organizational and research problems, as open democracy requires, may yield superior outcomes, by promoting those qualities presumed by trust (see [31], and [39] for a review of relevant evidence).

Current problems with the university environment that were identified earlier included the overuse of key performance indicators and the use of a top-down governance structure. Yet democracy as such is orthogonal to both specific decisions concerning working conditions (e.g. whether staff are incentivized by particular metrics), and also by whether the flow of control runs from top down or bottom up in terms of committee structure. A democratic university may (or may not) decide to incentivize, for example, grant income in promotion criteria. If they do, this will be a decision whose details will be finessed by those who daily confront what the real trade-offs are. And given general dissatisfaction with the way senior management attempts to incentivize academics now, a democratic university may in at least some institutions come to different decisions in detail. Importantly, the decision will be made by faculty knowing they are trusted to make decisions, because open democracy embodies Theory Y thinking. When metrics are decided locally and with light touch by the experts, and used with judgement as a means and not an end, they can be useful and need not be demotivating [39].

Similarly, the flow of control can still be democratically top down as in figure 3, allowing greater coherence across the university (democratic decisions can apply to the whole university), or bottom up, allowing different schools more independence. The two directions of flow can both be democratic because the people at the bottom and the top are, over time, the same when there is assortment by lot, or the use of deliberative polls. What the direction of the flow of control is for a given institution can be worked out according to the needs of a particular institution (say, by a deliberative poll). And in whichever direction control goes, once again a crucial difference remains compared with the status quo: An open democratic system embodies Theory Y thinking.

Once a democratic organizational structure is in place, the decisions that result can be jointly owned. It will be us who made the decisions. We can say: this is our house, we built it. Our state. We as citizens may make a mess of it, as we invariably must, as any decision-making procedure will. But it will be our mess, our problems to solve—together.

## Data Availability

This article has no additional data.

## Footnotes

1 Leaf [33] describes widely varying levels of democratic governance across different US Universities, yet indicates the direction of change is toward less democracy. For the rise of managerialism in US Universities, see Ginsberg [34]. For the variability in extent of democratic governance across Europe, but the same direction of change as the UK, see de Boer & File [35]. And for the headlong embrace of managerialism by Australian Universities, see Biggs [36] and Hil [37].

2 For the greater efficiencies that would be achieved by grant lotteries, see Gross & Bergstrom [43]. For a different solution, see Brette [44].

3 Again, this finding is consistent with some papers in high-impact-factor journal being rigorous. For example, plausibly if the papers are Registered Reports, then they may have good methodology (e.g. [50]). But then consider the quality of Registered Reports in PCI RR, which is free to authors and readers, and whose rigour the reader can assess for themselves because the whole process is open: https://rr.peercommunityin.org/.

4 Information integration in different organizational structures could be tested by injecting information into an organization at different places and then at a later time measure whether that information has been used to make decisions at different points in the organizational structure; compare measuring information integration in the human brain [56].
